# C57BL/6 Mice Pretreated With Alpha-Tocopherol Show a Better Outcome of *Trypanosoma cruzi* Infection With Less Tissue Inflammation and Fibrosis

**DOI:** 10.3389/fimmu.2022.833560

**Published:** 2022-01-28

**Authors:** Amanda C. O. Silva, Maiara Bonfim, Jonathan L. M. Fontes, Washington L. C. dos-Santos, José Mengel, Fabíola Cardillo

**Affiliations:** ^1^ Laboratory of Molecular and Structural Pathology, Gonçalo Moniz Institute, Fiocruz, Salvador, Brazil; ^2^ Department of Pathology, Faculty of Medicine, Federal University of Bahia, UFBA, Salvador, Brazil; ^3^ Oswaldo Cruz Institute, Fiocruz, Rio de Janeiro, Brazil; ^4^ Petrópolis Medical School, UNIFASE, Petrópolis, Brazil

**Keywords:** Chagas’ disease, *Trypanosoma cruzi*, fibrosis, immunomodulation, adjuvants, interleukin-10, interferon-γ, inflammation

## Abstract

Chagas disease is accompanied by a multisystem inflammatory disorder that follows *Trypanosoma cruzi* infection. Alpha-tocopherol has been described as an antioxidant and a potential adjuvant to enhance immune responses to vaccines. Therefore, we have evaluated the immune response to *T. cruzi* infection upon alpha-tocopherol pre-administration. The results show that administration of alpha-tocopherol before the infection results in lower parasitemia and lower mortality of C57BL/6 mice infected with the *Tulahuen T. cruzi* strain. Alpha-tocopherol administration in normal C57BL/6 mice resulted in higher levels of IFN-γ production by T and NK cells before and after the infection with *T. cruzi*. More importantly, previous administration of alpha-tocopherol increased the production of IL-10 by T and myeloid suppressor cells and the formation of effector memory T cells while decreasing the expression of PD-1 on T cells. These results suggest that alpha-tocopherol may limit the appearance of dysfunctional T cells during the acute and early chronic phases of *T. cruzi* infection, contributing to control infection. In addition, alpha-tocopherol could diminish tissue inflammation and fibrosis in late acute disease. These results strongly suggest that alpha-tocopherol may be a helpful agent to be considered in Chagas disease.

## Introduction

*Trypanosoma cruzi* is the etiological agent of Chagas disease or American Trypanosomiasis (1). It is estimated that approximately 6 to 7 million people are infected worldwide, mainly in Latin America. Later, the disease does appear on other continents (2). Furthermore, 100 million people are at risk of contamination, and 10000 deaths annually can be attributed to the infection (3). Chagas disease is a significant public health problem worldwide (2, 4).

The disease is characterized by two distinct phases: acute and chronic. In the acute phase, trypomastigotes are detected in the peripheral blood. The acute infection may be asymptomatic in humans (5). However, general symptoms like fever, tachycardia, mild splenomegaly and adenomegaly may be found (6). Trypomastigote forms can invade many organs and tissues, but the usual targets are different muscle tissue types, macrophages, and neurons (7).

After infection, a strong immune response is built to control the parasite numbers (4). The control of *T. cruzi* acute infection requires activating and establishing several innate and adaptative effector mechanisms. The roles of the immune system in the dynamics of the disease include containment of parasitic replication, control of the propagation of the parasite in target tissues, tissue inflammation, and its regulation afterward (8). The inflammatory process is crucial in establishing severe disease (8, 9).

NK cells, neutrophils, dendritic cells, and macrophages are important components of the innate response during acute infection (10). Antibodies and cytokines produced by B lymphocytes and the activation of CD4^+^, CD8^+^, γδ^+^, and NK^+^ T lymphocytes are critical for infection control and progression to the chronic phase (8, 10).

In addition to cells, pro-inflammatory cytokines may be essential during the acute *T. cruzi* infection. The cytokines associated with the phenotype of resistance are interferon-γ (IFN-γ) (11), interleukin-12 (IL-12), and tumor necrosis factor-α (TNF) (12). IFN-γ participates in activating phagocytic cells, stimulating these cells to produce reactive oxygen species (ROS) and nitric oxide and polarizing the CD4 T lymphocyte response to a Th1 profile (13).

Other studies reinforce the role of interleukin-10 (IL-10) as a cytokine with activity related to the profile of resistance and protection against uncontrolled immune response during immune responses against protozoan infections (14, 15). In this case, the immune response is down-modulated by IL-10, preventing the pathology from occurring. IL-10 may also help control acute infection, as previously demonstrated (16).

Presently, a couple of drugs are used to treat the acute Chagas disease, and they appear to have no great effects on established conditions (17). Also, vaccines are unavailable, and there are no alternative treatment options currently available. Therefore, strategies or potential drugs are highly desirable to diminish inflammation and fibrosis during the infection (18).

Alpha-tocopherol is an isomer of vitamin E, described as the most potent biological form among the isomers (19). Vitamin E has been shown to have an adjuvant effect when conjugated with antigens, as in the study by Karlsson et al. (20). This study evaluated the vaccination in mice with plasmids encoding proteins for the influenza virus using alpha-tocopherol as an adjuvant. The results indicated a more potent and balanced response to IgG1 and IgG2c using plasmids together with alpha-tocopherol (20). Furthermore, immunization with tetanus toxoid and alpha-tocopherol increased specific anti-tetanus toxin antibodies and splenocyte levels of IFN-γ and IL-4 compared to a conventional adjuvant (21). In addition, alpha-tocopherol is an immunomodulatory compound, helping to control tumor size in many different models (22–24). Vitamin E has also shown *in vitro* biological activity. For instance, mouse splenic T lymphocytes cultured in the presence of vitamin E had an increased cell proliferation and IL-2 production (25).

Vitamin E deficiency is associated with sepsis in children and might contribute to the development of septic shock during different bacterial infections (26). One study showed myocarditis aggravation and augmented sympathetic heart denervation in vitamin E-deficient rats during *T. cruzi* infection (27). Therefore, suggesting that vitamin E could be beneficial to control the inflammatory condition found in the *T. cruzi* infection.

As *T. cruzi* infection causes an exacerbated immune response with tissue damage in the host, the present study sought to investigate whether pre-treatment with alpha-tocopherol can promote the control of pro-inflammatory conditions during acute infection. We have demonstrated that previous alpha-tocopherol administration can induce a better control of *T. cruzi* infection and efficiently modulate the immune response in tissues, such as the heart and skeletal muscles. This study indicates that alpha-tocopherol might help promote a better disease evolution.

## Materials and Methods

### Animals

C57BL/6 mice 4 to 6 weeks old, bred and maintained in the Animal Facility of the Gonçalo Moniz Institute (IGM), Fiocruz, Bahia. All experiments were conducted according to protocols approved by the Animal Use Ethics Committee (CEUA L-IGM-007/2017) of the IGM.

### Experimental Groups

Experimental groups were divided according to the intervention performed. The groups were: Control, with no intervention; Veh/Infected: animals that received only the vehicle and were infected or Aphatoc/Infected (treated only with alpha-tocopherol) before the infection. The administration scheme of these components in the different groups was one dose per week, subcutaneously, for one month. Groups were infected after 30 days from the last injection.

### Treatment With Alpha-Tocopherol

Alpha-tocopherol (Sigma-Aldrich, cat# T3634) was administrated subcutaneously before infection with *T. cruzi*. The alpha-tocopherol was diluted in DMSO, and the solution was kept in an ultrasound bath to help homogenization. New solutions were prepared before use. The volume per injection was adjusted for a dose of 100 mg of alpha-tocopherol/Kg/injection/animal (28). Different organs were analyzed for acute or chronic alphatoc toxicity, using the dose/scheme described above, and no signs of toxicity were found (data not shown).

### Parasites and Infection

*T. cruzi* Tulahuen strain was used (29). The parasites were maintained by serial passage in mice before inoculation. The infection of the experimental groups was carried out as previously described (30). Mice were injected intraperitoneally with 50 or 1x10^3^ trypomastigotes. Mice were euthanized and evaluated 60 days post-infection (dpi).

### Determination of Parasitemia

The number of circulating parasites was determined on different days post-infection. Counting was performed under an optical microscope, evaluating the number of parasites in 100 microscopic fields (40x magnification). 5 µL of blood was collected from the animals’ tails (30).

### Cell Cultures

Spleens from animals in the different experimental groups were aseptically removed, washed, and resuspended to determine the number of cells per milliliter. Splenocytes were plated in 24-well culture plates. Each well contained 5x10^6^ cells/mL, which were incubated in RPMI containing 10% FBS in a 5% CO_2_ at 37°C. Brefeldin-A was added 4 hours before the cells were harvested to stain them for flow cytometric analysis. After 12 hours of culture, the cells were plated in 96-well plates containing 2x10^6^ cells in each 100 μL. Subsequently, the plates were centrifuged at 2000 rpm for 3 seconds and cells stained.

### Flow Cytometric Analysis

Cells from the spleen and skeletal muscle were resuspended in FACS buffer (PBS, 2% FBS, and 0,1% sodium azide). Aliquots of the cell suspensions were incubated for 20 minutes and protected from light, with the following mAbs: anti-CD11b (Clone M1/70) FITC, anti-TCR αβ (Clone B20.1) FITC, anti-CD8 (Clone 53-6.7) PerCP-Cy 5.5 or FITC, anti-CD4 (Clone GK1.5) PerCP-Cy 5.5 or FITC, anti-Gr1 (Clone RB6-8C5) Pe Cy 5.5, anti-NK1.1 (Clone PK-136) Pe-Cy 5.5, anti-CD62L (Clone DREG-56) PE, anti-PD1 (Clone J43.1). PE-labeled mAb were from Invitrogen or eBioscience. A rat anti-mouse isotypes were also used as controls. For intracellular cytokine-staining, the previously stained cells were fixed with 1% paraformaldehyde for one hour and washed three times in BD Perm/Wash buffer. The intracellular cytokine staining was performed with anti-IFN-γ (Clone XMG1.2, Invitrogen), anti-IL10 (Clone JES5-16E3, eBioscience). Antibodies were diluted in Perm/Wash buffer, and the cells were incubated for 1 hour. They were washed twice with Perm/Wash and once more with FACS buffer. A BD LSRFortessa or FACScan instruments were used for readings.

### Gating Strategies for the Flow Cytometry Analysis

To obtain cytokine-producing CD4^+^ and CD8^+^ T cells, lymphocytes were gated in FSC x SSC plots. CD4^+^ or CD8^+^ T cells were identified in FL3 or FL1, respectively. Then, cytokine-producing cells were studied in FL2. Cytokine-producing MDSCs were gated and analyzed as follows: Gr1^high^ cells were identified in FL1 and CD11b^high^ in FL3. Gated Gr1^high^CD11b^high^ MDSCs were examined in FL2 for the presence of IL-10. A total of 200.000 events were collected per sample. Results were analyzed using Flowjo software.

### Histopathological and Quantitative Morphological Studies

The skeletal muscle and heart fragments were collected and fixed in Milloning formalin, embedded in paraffin to obtain 5 µm sections, stained in Hematoxylin and Eosin (H&E) or by Picrosirius red method. Later, the equipment photographed the slides at a maximum magnification of 200X (Virtual Slide Microscopy VS120, Olympus, Japan). Five images of each slide were taken, corresponding to an animal sample. The ImageJ Pro Plus software (computer) was used for the analyses. The morphometric determination of the inflammatory infiltrate considered the cell count throughout the image and subsequently calculated the total area of ​​the tissue. The total number of cells was divided by the total tissue area in mm^2^, and the value was represented by cells/mm². The images were also analyzed for fibrosis analysis using the ImageJ software with the Threshold Color plug-in. The areas based on color were selected and measured to quantify the percentage of collagen marked in red. Afterward, the total tissue area was measured, and then the percentage of collagen area in the entire area was calculated, as evidenced by “Pale pink” (Picrosirius red).

### Statistical Analysis

The Kolmogorov-Smirnov test was used to verify the normality of the sample distribution, using IBM SPSS Statistics (IBM) software. Other studies were performed using GraphPad Prism software, version 6.0. The samples then had a non-normal distribution, and the Mann-Whitney test was used. For analysis of mortality progression, the Log-rank test was used. P values less than 0.05 were considered significant.

## Results

### Alphatoc Administration Induces a Better Control of Parasitemia and Increases the Resistance to Infection

**Figure 1:**
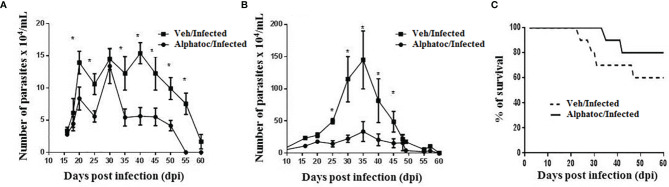
Parasitemia and survival of animals infected with the Tulahuen strain of *T. cruzi*. In **(A)**, an inoculum of 50 trypomastigote forms was used. The Alphatoc/Infected group was compared to the Vehicle (Veh)/Infected group (p=0.0238, day 20; p=0.0238, day 25; p=0.0238, day 35; p=0.0159, day 40; p=0.0238, day 45; p=0.0238, day 50; p=0.0079, day 55), Mann Whitney test, n=10. In **(B)**, mice were infected with 10^3^ forms of parasites and the Alphatoc/Infected group was again compared to the Veh/Infected group (p=0,0079, day 25; p=0,0159, day 30; p=0,0397, day 35; p=0,0476, day 40; p=0,0476, day 45), Mann Withney test, n=10 at the beginning of the experiments). In **(C)**, Results represent the survival curves for infection with 10^3^ trypomastigote forms. Differences between the two experimental groups are indicated (Log-rank (Mantel-Cox) test, P=0.0182, n=10 at the beginning of the experiment). The asterisk (*) shows statistically significant differences. Data are for one representative experiment out of two similar experiments.

C57BL/6 mice were previously treated with alphatoc and then infected with 50 or 1x10^3^ trypomastigotes (**Figure 1**). **Figures 1A, B** show lower parasitemia in mice treated with alphatoc than in the untreated infected group of mice at many time points after initial infection. **Figure 1C** shows higher survival in the alphatoc-pretreated infected mice than in the untreated control. The results show a 60% mortality rate in the vehicle-pretreated infected animals, and only 20% of alphatoc-pretreated and infected mice died within 60 days post-infection. No mortality was recorded when 50 forms were used to infect the mice in either group. Thus, alphatoc treatment before the infection induces higher resistance to these animals.

### Alphatoc Pre-Treatment Increases the Numbers of IFN-γ-Producing Cells in Both Infected and Uninfected Animals

**Figure 2:**
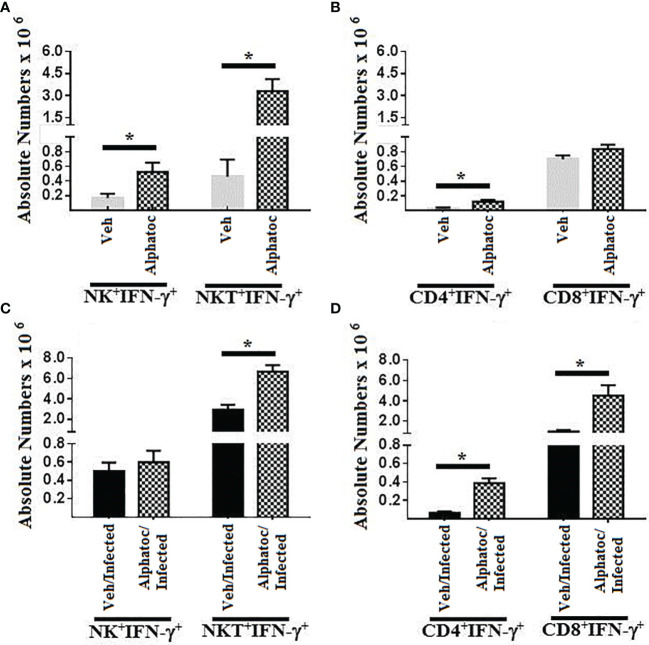
IFN-γ production by NK and T cell splenocytes. In **(A)**, IFN-γ-producing NK and NKT cells in uninfected animals pretreated with vehicle or alphatoc were compared. **(B)**, CD4 or CD8 T cell splenocytes producing IFN-γ are shown in the vehicle or alphatoc pretreated animals. In **(C)**, NK and NKT cells producing IFN-γ are shown. The alphatoc/infected group was individually compared to the other groups. In **(D)**, the alphatoc/infected group was individually compared to the other groups (*P < 0,05, Mann Whitney test, n=4). Data are for one representative experiment out of three.

**Figures 2A, C** show the total numbers of splenic NK^+^ and NKT^+^ cells producing interferon-γ in uninfected mice (**Figure 2A**) or infected animals (**Figure 2C**). Pre-treatment with vehicle or alphatoc is indicated. Splenic numbers of NK^+^IFN-γ^+^ and NKT^+^IFN-γ^+^ cell numbers were augmented when uninfected mice were pretreated with alphatoc compared to vehicle-treated mice. However, no difference in the splenic numbers of NK^+^IFN-γ^+^ cells was detected after infection (**Figure 2C**). Also, the total numbers of splenic NKT^+^IFN-γ^+^ cells were increased in the group previously treated with alphatoc (**Figure 2C**). **Figure 2B** shows the whole numbers of CD4^+^IFN-γ^+^ and CD8^+^IFN-γ^+^ splenic T cells in uninfected animals pretreated or not with alphatoc. The results show that alphatoc pre-administration augmented the total splenic CD4^+^ but not CD8^+^ T cells expressing IFN-γ in mice pretreated with vehicle. Infected groups pretreated with alphatoc had higher numbers of CD4^+^ and CD8^+^ T cells producing IFN-γ than vehicle pretreated and infected mice (**Figure 2D**).

### Infected Mice Pretreated With Alphatoc Present More Effector Memory Splenic CD8^+^ T Cells

**Figure 3:**
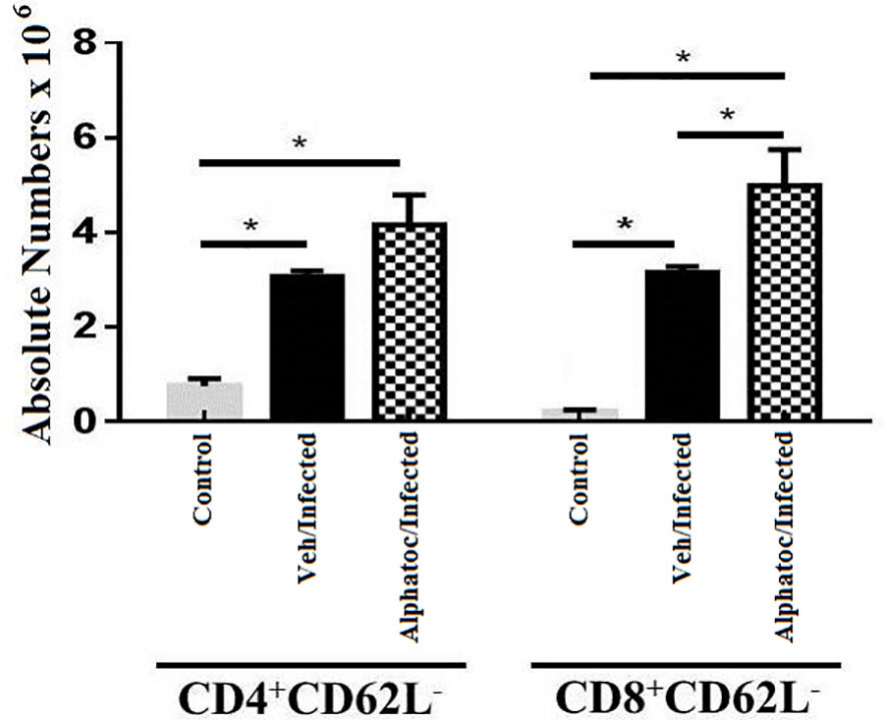
Quantification of splenic effector memory splenic T cells. Absolute numbers of splenic effector memory T cells in (CD4^+^CD62L^-^ or CD8^+^CD62L^-^ after 60 days of infection with 10^3^ forms are shown (*P < 0,05, Mann Whitney test, n=4). Vehicle and alphatoc pretreated and infected groups were compared. A third untreated group was used as a control. Data are for one representative experiment out of three.

The expression of L-selectin (CD62L) was studied to analyze the effector profile of CD4 and CD8 splenic T lymphocytes. Sixty days after *T. cruzi* infection with 1000 forms of trypomastigotes, the alphatoc/infected and vehicle/infected groups presented a higher number of splenic CD4^+^CD62L^neg^ cells when compared to the control groups (**Figure 3**). However, effector memory CD8^+^CD62L^neg^ T cells were higher in infected mice previously treated with alphatoc than in the other experimental groups (**Figure 3**).

### PD-1 Expression in Splenic T Cells of Alphatoc or Vehicle Pretreated and Infected Mice

**Figure 4:**
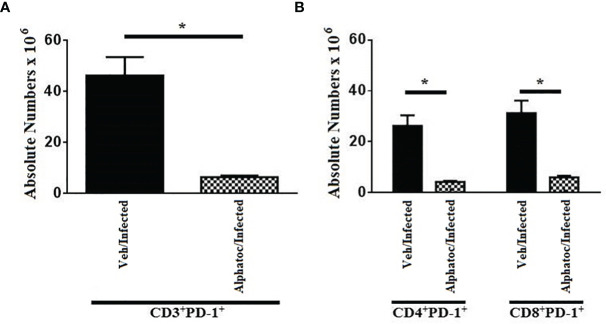
PD-1 expression in CD3^+^, CD4^+^ and CD8^+^ splenic T cells from animals on day 60^th^ after infection with 10^3^ forms. In **(A)**, absolute numbers of CD3^+^PD1^+^ are shown. In **(B)**, total numbers of CD4^+^PD1^+^ and CD8^+^PD1^+^ T cells are depicted. Groups of mice pretreated with vehicle or alphatoc were infected and individually compared after 60 days (*P < 0,05, Mann Whitney test, n= 4). A third untreated group was used as a control. Data are for one representative experiment out of two.

As we detected an increased production of interferon-γ by T cells and an augmented formation of effector T cells upon alphatoc pre-administration, we evaluated the expression of the PD-1 molecule, a marker for dysfunctional or anergic T cells, in mice pretreated with vehicle or alphatoc and infected afterward. After 60 days of infection, both the total numbers of CD3^+^PD-1^+^, CD4^+^PD-1^+^, and CD8^+^PD-1^+^ T cells were decreased in the alphatoc/infected compared to the veh/infected mice (**Figures 4A, B**), thus denoting that alphatoc may prevent T cell disfunction/anergy during the acute *T. cruzi* infection.

### Alphatoc Pre-Treatment Diminished Tissue Inflammation and Fibrosis in the Late Acute *T. cruzi* Infection

**Figure 5:**
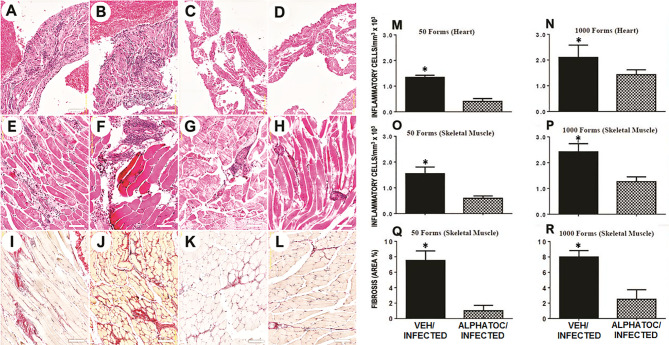
Morphometric analysis and fibrosis of inflammatory infiltrate after 60 days of infection with 50 or 10^3^ forms of *T. cruzi*. Representative images of the histological lesions are depicted in figures **(A–L)**. Morphometric analyses of the inflammatory infiltrate and fibrosis area are shown in figures **(M–R)**. Heart atrium is shown in figures **(A–D)**. Skeletal muscle is depicted in figures **(E–H)**. Figures **(I–L)** show representative images of the skeletal muscle fibrosis. All the histological analysis was performed in C57BL/6 mice after 60 days of infection with 50 **(A**, **C**, **E**, **G**, **I**, **K)** or 10^3^ **(B**, **D**, **F**, **H**, **J**, **L)** trypomastigotes. Mice were pretreated with vehicle alone **(A**, **B**, **E**, **F**, **I**, **J)** or alphatoc **(C**, **D**, **G**, **H**, **K**, **L)**. The groups with significant differences are represented with (*), Mann Whitney test, n= 4, P<0.05.

Sixty days after the infection, skeletal and cardiac muscles were evaluated by histology (**Figure 5**). Representative photomicrographs (HE staining) from mice infected with 50 (**Figures 5A, C, E, G, I, K**) or 10^3^ (**Figures 5B, D, F, H, J, L**) trypomastigotes are shown. After infection, vehicle-treated animals presented a diffuse inflammatory infiltrate in the atrium (**Figures 5A, B**). In alphatoc pretreated mice, a reduced inflammatory cell infiltrate was found in the atrium (**Figures 5C, D**).

In infected vehicle pretreated animals, inflammation spread among skeletal, muscular fibers (**Figures 5E, F**). In alphatoc pretreated animals (**Figures 5G, H**), inflammatory infiltrate was restricted to the perivascular interstitium. Interstitial fibrosis (Picrosirius red staining) was evident in the vehicle pretreated animals (**Figures 5I, J**) and was minimal or absent in alphatoc pretreated animals (**Figures 5K, L**). Thus demonstrating that mice treated with alphatoc before the infection are more protected against tissue lesions than untreated mice.

Quantitative studies show the number of inflammatory cells in the heart or skeletal muscle sections from alphatoc-treated or vehicle-treated infected mice, as indicated in **Figures 5M–P**, respectively. The results show that pre-treatment with alphatoc led to diminished heart or skeletal muscle inflammatory cells. Despite the inoculum, fibrosis areas showed a considerable reduction of collagen deposition in skeletal muscle from mice pretreated with alphatoc (**Figures 5Q, R**).

### Alphatoc Pre-Administration Increased the Numbers of Splenic CD8^+^ T Cells and Myeloid Suppressor Cells Expressing IL-10

**Figure 6:**
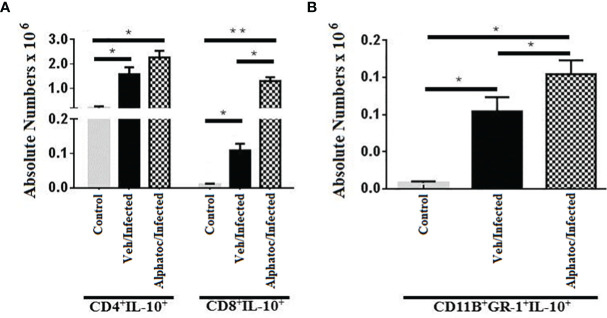
Numbers splenic T cells and MDSCs, expressing IL-10. **(A)** shows the numbers of IL-10 producing CD4^+^ and CD8^+^ splenic T lymphocytes after 60 days of infection with 10^3^ forms of *T. cruzi*. IL-10-producing Gr1^+^CD11b^+^ (MDSC) splenic cells are shown in **(B)**. All groups were individually compared (**P < 0,01, *P < 0,05, Mann Whitney test, n= 4).

Because alphatoc pre-treatment resulted in a more benign acute infection and less tissue inflammation, IL-10, a cytokine with anti-inflammatory functions, was studied. **Figure 6A** shows the number of splenic CD8^+^IL-10^+^ increased significantly in animals pretreated with alphatoc. In addition, the number of splenic myeloid suppressor cells able to produce IL-10 (CD11b^+^GR-1^+^IL-10^+^) also rose on day 60^th^ post-infection (**Figure 6B**). The percentages (not shown) and numbers of CD4^+^IL-10^+^ T cells were not different from infected vehicle pretreated mice (**Figure 6A**).

## Discussion

*T. cruzi* infection induces pro-inflammatory responses followed by robust and persistent immune responses that may culminate in immunopathology and progress to more severe disease. In this context, we sought to assess whether alpha-tocopherol could alter the profile of regulatory, pro-inflammatory, or specific responses that help modulate the illness in the initial chronic phase of *T. cruzi* infection.

Over time, several experimental models were used to understand the dynamics of cell populations during experimental *T. cruzi* infection. C57BL/6 are susceptible to infection by the Tulahuen strain, with high mortality rates, especially when injected with a high inoculum of *T. cruzi* (29, 31).

The present study investigated the number of circulating parasites and mortality of animals of the C57BL/6 lineage infected with the Tulahuen strain of *T. cruzi*, previously treated with alphatoc. The animals were infected with 50 or 10^3^ trypomastigote forms (**Figures 1A, B**, respectively). The results show that parasitemia levels were lower in the groups treated with alphatoc than untreated. There was no mortality in the groups infected with 50 forms regardless of the treatment (data not shown). In infected mice with a higher inoculum parasite (10^3^), the longevity of animals treated with alpha-tocopherol and infected was 80% compared to only 40% of survival in untreated mice (**Figure 1C**). In addition, to describe alphatoc’s effect on parasitemia and survival, we have evaluated what could be associated with increased resistance in these alphatoc pretreated animals.

The parasitemia control and increased survival may be associated with the augmented functional activity of NK and T cells in the acute phase of infection, as previously shown in other studies (32–34). Alphatoc pre-treatment induced the expression of IFN-γ and increased the numbers of NK and T cells after its administration before the infection (**Figures 2A, B**). The increased numbers of NKT, CD4^+^, and CD8^+^ cells were kept up to day 60th after inoculum, indicating a long-term effect (**Figures 2C, D**). Some studies suggest that the amount of memory T cells before the infection and the quantity of memory T cells may also correlate with resistance (29, 35). The relevance of effector T cells for protection during acute illness may be associated with a better capacity to produce cytokines such as IFN-γ and, therefore, activation of microbicidal mechanisms of phagocytic cells (36–38). Consequently, we have evaluated the numbers of splenic effector memory T cells on day 60 after infection. **Figure 3** shows increased splenic effector memory CD8^+^ T cells in mice pretreated with alphatoc compared to the vehicle pretreated group. Memory CD8^+^ T cells are crucial in infection control, especially in the infected tissues (38, 39). However, it was also described that memory T cells might acquire a non-functional phenotype in peripheral tissues either in tumor environments or during *T cruzi* infection, the so-called exhausted or dysfunctional phenotype (40, 41). Therefore, the simple presence of increased numbers of effector memory T cells does not guarantee a better immune response. A marker usually expressed in exhausted or dysfunctional T cells is the PD-1 molecule (42). Therefore, PD-1 expression was evaluated in splenic T cells from mice treated with alphatoc previously to infection (**Figures 4A, B**). The results showed that the expression of PD-1 was greatly reduced in T cells from mice pretreated with alphatoc, thus indicating that alphatoc is inhibiting the generation of exhausted or dysfunctional T cells during the acute *T. cruzi* infection.

In the acute *T. cruzi* infection, treatment with monoclonal antibodies anti-PD1 in C57BL/6 mice can improve the cellular immune response and decrease the parasite load in the heart (43, 44). It has been shown that the high expression of PD-1 contributes to the evasion and establishment of parasites (43–45). Our results indicate that *in vivo* administration of alphatoc may induce a better infection outcome, increasing the production of IFN-γ, effector memory T cells, and inhibiting the production of exhausted or dysfunctional T cells. Also, the mechanism responsible for this biological effect is unknown.

It was also described that PD-1 blocking during the acute phase of *T. cruzi* infection might increase tissue lesion despite a better parasite clearance (44). Therefore we conducted histopathological evaluations in animals infected with 50 or 10^3^ trypomastigotes. These studies revealed that the group that received alphatoc had fewer inflammatory cells in the heart and skeletal muscle (**Figure 5**). Fibrosis was reduced in the alphatoc/infected group regardless of inoculum (**Figure 5**). In conclusion, the main benefits and treatments with alphatoc before *T. cruzi* infection were reducing parasitemia, increasing survival, and reducing complications generated in the target tissues, such as intense inflammatory infiltrate and fibrosis.

Corroborating that regulatory mechanisms are active in alphatoc pretreated animals, an increased number of CD8^+^ T cells and MDSCs, expressing IL-10 were found (**Figures 6A, B**). The increase in IL-10 can promote a more balanced immune response in these animals, keeping the levels of pro-inflammatory cytokines within ranges compatible with infection control and regulating the inflammation caused by IL-12, IFN-γ, and TNF (12, 15, 46). IL-10 knockout mice infected with *T. cruzi* show increased susceptibility to lethal infection and more significant pathogenic responses associated with pro-inflammatory cytokines (47). In addition, these animals showed early mortality and more significant inflammation than wild-type mice. As a counterproof, this study also showed that IL-10^-^/^-^ animals treated with recombinant IL-10 increased survival, had a delay in mortality, and a reduction in IFN-γ produced by CD4^+^ T cells.

Similarly, Holscher et al. (47) showed that C57BL/6 IL-10^-^/^-^, had a more vigorous production of pro-inflammatory cytokines. Therefore, there is a need for IL-10 to control a pathological immune response in the acute phase of infection. Yet, in the experiments described here, there was a rise in the production of IFN-γ and IL-10. This observation might reflect the activity of polyfunctional T cells that may secrete both cytokines simultaneously (48). The low levels of PD-1 expression may contribute to the appearance of polyfunctional T cells. In this case, it would be advantageous since IL-10 could prevent or diminish some of the harmful effects of high levels of inflammatory cytokines such as IFN-γ yet help resolve the infection.

We have shown herein that alpha-tocopherol pre-administration is associated with an improvement in the outcome of *T. cruzi* infection. A reduction in the inflammatory infiltrates and fibrosis in the heart and skeletal muscle was particularly impressive. However, the observed biological effects deal with the preventive aggravation of the disease, an experimental condition that may not be feasible in the real world. Still, it documents an important role for vitamin E as an immunomodulator for *T. cruzi* infection, suggesting a potential use during the acute and chronic phases of the disease. Yet, unpublished studies from our group show that alpha-tocopherol administration during the chronic phase of *T. cruzi* infection has similar effects, particularly when associated with *T. cruzi* antigens (manuscript in preparation).

## Data Availability Statement

The original contributions presented in the study are included in the article/supplementary material. Further inquiries can be directed to the corresponding authors.

## Ethics Statement

Ethical review and approval was not required for the animal study because the involvement of animals was solely for the harvesting of cells for culturing and *in vitro* experiments. This study was conducted in accordance with the ethical conduct in the care and use of animals and in compliance with ARRIVE guidelines.

## Author Contributions

AS, MB, FC, and JM contributed to conception, and design of the study. AS, MB, JF, WD-S, FC, and JM organized the database. AS and MB performed the statistical analysis. FC and JM wrote the first draft of the manuscript. FC, WD-S, and JM wrote sections of the manuscript. All authors contributed to manuscript revision, read, and approved the submitted version

## Funding

This work was supported by CNPq, FAPESB, FAPERJ, FIOCRUZ and FOG (Fundação Octácilio Gualberto).

## Conflict of Interest

The authors declare that the research was conducted in the absence of any commercial or financial relationships that could be construed as a potential conflict of interest.

The reviewer JLV declared a shared affiliation with several of the authors, WS, JM, FC, to the handling editor at the time of review.

## Publisher’s Note

All claims expressed in this article are solely those of the authors and do not necessarily represent those of their affiliated organizations, or those of the publisher, the editors and the reviewers. Any product that may be evaluated in this article, or claim that may be made by its manufacturer, is not guaranteed or endorsed by the publisher.

## References

[1] Chagas C. A New Disease Entity in Man: A Report on Etiologic and Clinical Observations. Int J Epidemiol (2008) 37(4):694–5. doi: 10.1093/ije/dyn149 18653502

[2] Lidani KCF Andrade FA Bavia L Damasceno FS Beltrame MH Messias-Reason IJ. Chagas Disease: From Discovery to a Worldwide Health Problem. Front Public Health (2019) 7:166. doi: 10.3389/fpubh.2019.00166 31312626 PMC6614205

[3] Stanaway JD Roth G. The Burden of Chagas Disease: Estimates and Challenges. Global Heart (2015) 10(3):139–44. doi: 10.1016/j.gheart.2015.06.001 26407508

[4] Rassi A Jr. Rassi A Marin-Neto JA. Chagas Disease. Lancet (2010) 375(9723):1388–402. doi: 10.1016/S0140-6736(10)60061-X 20399979

[5] Echavarria NG Echeverria LE Stewart M Gallego C Saldarriaga C. Chagas Disease: Chronic Chagas Cardiomyopathy. Curr Probl Cardiol (2021) 46(3):100507. doi: 10.1016/j.cpcardiol.2019.100507 31983471

[6] Clayton J. Chagas Disease 101. Nature (2010) 465(7301):S4–5. doi: 10.1038/nature09220 20571553

[7] Santi-Rocca J Fernandez-Cortes F Chillon-Marinas C Gonzalez-Rubio ML Martin D Girones N. A Multi-Parametric Analysis of Trypanosoma Cruzi Infection: Common Pathophysiologic Patterns Beyond Extreme Heterogeneity of Host Responses. Sci Rep (2017) 7(1):8893. doi: 10.1038/s41598-017-08086-8 28827716 PMC5566495

[8] Cardillo F de Pinho RT Antas PR Mengel J. Immunity and Immune Modulation in Trypanosoma Cruzi Infection. Pathog Dis (2015) 73(9):ftv082. doi: 10.1093/femspd/ftv082 26438729 PMC4626602

[9] Andrade ZA. Immunopathology of Chagas Disease. Mem Inst Oswaldo Cruz (1999) 94 Suppl 1:71–80. doi: 10.1590/s0074-02761999000700007 10677693

[10] Golgher D Gazzinelli RT. Innate and Acquired Immunity in the Pathogenesis of Chagas Disease. Autoimmunity (2004) 37(5):399–409. doi: 10.1080/08916930410001713115 15621564

[11] Cardillo F Voltarelli JC Reed SG Silva JS. Regulation of Trypanosoma Cruzi Infection in Mice by Gamma Interferon and Interleukin 10: Role of NK Cells. Infect Immun (1996) 64(1):128–34. doi: 10.1128/iai.64.1.128-134.1996 PMC173737 8557330

[12] Abrahamsohn IA Coffman RL. Trypanosoma Cruzi: IL-10, TNF, IFN-Gamma, and IL-12 Regulate Innate and Acquired Immunity to Infection. Exp Parasitol (1996) 84(2):231–44. doi: 10.1006/expr.1996.0109 8932773

[13] Gazzinelli RT Hieny S Wynn TA Wolf S Sher A. Interleukin 12 Is Required for the T-Lymphocyte-Independent Induction of Interferon Gamma by an Intracellular Parasite and Induces Resistance in T-Cell-Deficient Hosts. Proc Natl Acad Sci USA (1993) 90(13):6115–9. doi: 10.1073/pnas.90.13.6115 PMC46878 8100999

[14] Hunter CA Ellis-Neyes LA Slifer T Kanaly S Grunig G Fort M. IL-10 Is Required to Prevent Immune Hyperactivity During Infection With Trypanosoma Cruzi. J Immunol (1997) 158(7):3311–6. 9120288

[15] Couper KN Blount DG Riley EM. IL-10: The Master Regulator of Immunity to Infection. J Immunol (2008) 180(9):5771–7. doi: 10.4049/jimmunol.180.9.5771 18424693

[16] Roffe E Rothfuchs AG Santiago HC Marino AP Ribeiro-Gomes FL Eckhaus M. IL-10 Limits Parasite Burden and Protects Against Fatal Myocarditis in a Mouse Model of Trypanosoma Cruzi Infection. J Immunol (2012) 188(2):649–60. doi: 10.4049/jimmunol.1003845 PMC3253255 22156594

[17] Morillo CA Marin-Neto JA Avezum A Sosa-Estani S Rassi A Jr Rosas F. Randomized Trial of Benznidazole for Chronic Chagas’ Cardiomyopathy. N Engl J Med (2015) 373(14):1295–306. doi: 10.1056/NEJMoa1507574 26323937

[18] Hoffman KA Villar MJ Poveda C Bottazzi ME Hotez PJ Tweardy DJ. Signal Transducer and Activator of Transcription-3 Modulation of Cardiac Pathology in Chronic Chagasic Cardiomyopathy. Front Cell Infect Microbiol (2021) 11:708325. doi: 10.3389/fcimb.2021.708325 34504808 PMC8421853

[19] Lee GY Han SN. The Role of Vitamin E in Immunity. Nutrients (2018) 10(11). doi: 10.3390/nu10111614 PMC6266234 30388871

[20] Karlsson I Borggren M Nielsen J Christensen D Williams J Fomsgaard A. Increased Humoral Immunity by DNA Vaccination Using an Alpha-Tocopherol-Based Adjuvant. Hum Vaccin Immunother (2017) 13(8):1823–30. doi: 10.1080/21645515.2017.1321183 PMC5557247 28613978

[21] Radhakrishnan AK Mahalingam D Selvaduray KR Nesaretnam K. Supplementation With Natural Forms of Vitamin E Augments Antigen-Specific TH1-Type Immune Response to Tetanus Toxoid. BioMed Res Int (2013) 2013:782067. doi: 10.1155/2013/782067 23936847 PMC3722853

[22] dos Santos GA Abreu e Lima RS Pestana CR Lima AS Scheucher PS Thome CH. (+)Alpha-Tocopheryl Succinate Inhibits the Mitochondrial Respiratory Chain Complex I and Is as Effective as Arsenic Trioxide or ATRA Against Acute Promyelocytic Leukemia. Vivo Leukemia (2012) 26(3):451–60. doi: 10.1038/leu.2011.216 21869839

[23] Angulo-Molina A Reyes-Leyva J Lopez-Malo A Hernandez J. The Role of Alpha Tocopheryl Succinate (Alpha-TOS) as a Potential Anticancer Agent. Nutr Cancer (2014) 66(2):167–76. doi: 10.1080/01635581.2014.863367 24364743

[24] Freitas RA Silva dos Santos GA Gimenes Teixeira HL Scheucher PS Lucena-Araujo AR Lima AS. Apoptosis Induction by (+)Alpha-Tocopheryl Succinate in the Absence or Presence of All-Trans Retinoic Acid and Arsenic Trioxide in NB4, NB4-R2 and Primary APL Cells. Leukemia Res (2009) 33(7):958–63. doi: 10.1016/j.leukres.2008.09.035 19013639

[25] Adolfsson O Huber BT Meydani SN. Vitamin E-Enhanced IL-2 Production in Old Mice: Naive But Not Memory T Cells Show Increased Cell Division Cycling and IL-2-Producing Capacity. J Immunol (2001) 167(7):3809–17. doi: 10.4049/jimmunol.167.7.3809 11564798

[26] Dang H Li J Liu C Xu F. The Association Between Vitamin E Deficiency and Critically Ill Children With Sepsis and Septic Shock. Front Nutr (2021) 8:648442. doi: 10.3389/fnut.2021.648442 34222298 PMC8241937

[27] Carvalho LS Camargos ER Almeida CT Peluzio Mdo C Alvarez-Leite JI Chiari E. Vitamin E Deficiency Enhances Pathology in Acute Trypanosoma Cruzi-Infected Rats. Trans R Soc Trop Med Hygiene (2006) 100(11):1025–31. doi: 10.1016/j.trstmh.2005.12.009 16620891

[28] Manosso LM Neis VB Moretti M Daufenbach JF Freitas AE Colla AR. Antidepressant-Like Effect of Alpha-Tocopherol in a Mouse Model of Depressive-Like Behavior Induced by TNF-Alpha. Prog Neuropsychopharmacol Biol Psychiatry (2013) 46:48–57. doi: 10.1016/j.pnpbp.2013.06.012 23816813

[29] Cardillo F Cunha FQ Tamashiro WM Russo M Garcia SB Mengel J. NK1.1+ Cells and T-Cell Activation in Euthymic and Thymectomized C57Bl/6 Mice During Acute Trypanosoma Cruzi Infection. Scandinavian J Immunol (2002) 55(1):96–104. doi: 10.1046/j.1365-3083.2002.01034.x 11841697

[30] Melo RC Brener Z. Tissue Tropism of Different Trypanosoma Cruzi Strains. J Parasitol (1978) 64(3):475–82. doi: 10.2307/3279787 96243

[31] Roggero E Perez A Tamae-Kakazu M Piazzon I Nepomnaschy I Wietzerbin J. Differential Susceptibility to Acute Trypanosoma Cruzi Infection in BALB/c and C57BL/6 Mice Is Not Associated With a Distinct Parasite Load But Cytokine Abnormalities. Clin Exp Immunol (2002) 128(3):421–8. doi: 10.1046/j.1365-2249.2002.01874.x PMC1906265 12067296

[32] Antunez MI Cardoni RL. Trypanosoma Cruzi: The Expansion of NK, T, and NKT Cells in the Experimental Infection. Exp Parasitol (2004) 106(3-4):85–94. doi: 10.1016/j.exppara.2004.03.008 15172215

[33] Sathler-Avelar R Vitelli-Avelar DM Teixeira-Carvalho A Martins-Filho OA. Innate Immunity and Regulatory T-Cells in Human Chagas Disease: What Must be Understood? Mem Inst Oswaldo Cruz (2009) 104 Suppl 1:246–51. doi: 10.1590/s0074-02762009000900031 19753480

[34] Sardinha LR Elias RM Mosca T Bastos KR Marinho CR D’Imperio Lima MR. Contribution of NK, NK T, Gammaand Alpha Beta T Cells to the Gamma Interferon Response Required for Liver Protection Against Trypanosoma Cruzi. Infect Immun (2006) 74(4):2031–42. doi: 10.1128/IAI.74.4.2031-2042.2006 PMC1418886 16552032

[35] Cardillo F Nomizo A Postol E Mengel J. NK1.1 Cells Are Required to Control T Cell Hyperactivity During Trypanosoma Cruzi Infection. Med Sci Monitor: Int Med J Exp Clin Res (2004) 10(8):BR259–67. 15277986

[36] Gazzinelli RT Oswald IP Hieny S James SL Sher A. The Microbicidal Activity of Interferon-Gamma-Treated Macrophages Against Trypanosoma Cruzi Involves an L-Arginine-Dependent, Nitrogen Oxide-Mediated Mechanism Inhibitable by Interleukin-10 and Transforming Growth Factor-Beta. Eur J Immunol (1992) 22(10):2501–6. doi: 10.1002/eji.1830221006 1396957

[37] Tzelepis F de Alencar BC Penido ML Gazzinelli RT Persechini PM Rodrigues MM. Distinct Kinetics of Effector CD8+ Cytotoxic T Cells After Infection With Trypanosoma Cruzi in Naive or Vaccinated Mice. Infect Immun (2006) 74(4):2477–81. doi: 10.1128/IAI.74.4.2477-2481.2006 PMC1418894 16552083

[38] Tarleton RL. CD8+ T Cells in Trypanosoma Cruzi Infection. Semin Immunopathol (2015) 37(3):233–8. doi: 10.1007/s00281-015-0481-9 PMC4654118 25921214

[39] Keir ME Francisco LM Sharpe AH. PD-1 and Its Ligands in T-Cell Immunity. Curr Opin Immunol (2007) 19(3):309–14. doi: 10.1016/j.coi.2007.04.012 17433872

[40] Keir ME Butte MJ Freeman GJ Sharpe AH. PD-1 and Its Ligands in Tolerance and Immunity. Annu Rev Immunol (2008) 26:677–704. doi: 10.1146/annurev.immunol.26.021607.090331 18173375 PMC10637733

[41] Natale MA Minning T Albareda MC Castro Eiro MD Alvarez MG Lococo B. Immune Exhaustion in Chronic Chagas Disease: Pro-Inflammatory and Immunomodulatory Action of IL-27 *In Vitro* . PloS Negl Trop Dis (2021) 15(6):e0009473. doi: 10.1371/journal.pntd.0009473 34061845 PMC8195349

[42] Perez-Anton E Egui A Thomas MC Simon M Segovia M Lopez MC. Immunological Exhaustion and Functional Profile of CD8(+) T Lymphocytes as Cellular Biomarkers of Therapeutic Efficacy in Chronic Chagas Disease Patients. Acta Trop (2020) 202:105242. doi: 10.1016/j.actatropica.2019.105242 31669531

[43] Gutierrez FR Mariano FS Oliveira CJ Pavanelli WR Guedes PM Silva GK. Regulation of Trypanosoma Cruzi-Induced Myocarditis by Programmed Death Cell Receptor 1. Infection Immun (2011) 79(5):1873–81. doi: 10.1128/IAI.01047-10 PMC3088162 21357717

[44] Fonseca R Salgado RM Borges da Silva H Nascimento R D’Império-Lima MR Alvarez JM. Programmed Cell Death Protein 1–PDL1 Interaction Prevents Heart Damage in Chronic Trypanosoma Cruzi Infection. Front Immunol (2018) 9:997. doi: 10.3389/fimmu.2018.00997 29867974 PMC5949529

[45] Roy S Saha S Gupta P Ukil A Das PK. Crosstalk of PD-1 Signaling With the SIRT1/FOXO-1 Axis During the Progression of Visceral Leishmaniasis. J Cell Sci (2019) 132(9). doi: 10.1242/jcs.226274 30910830

[46] Moore KW de Waal Malefyt R Coffman RL O’Garra A. Interleukin-10 and the Interleukin-10 Receptor. Annu Rev Immunol (2001) 19:683–765. doi: 10.1146/annurev.immunol.19.1.683 11244051

[47] Holscher C Mohrs M Dai WJ Kohler G Ryffel B Schaub GA. Tumor Necrosis Factor Alpha-Mediated Toxic Shock in Trypanosoma Cruzi-Infected Interleukin 10-Deficient Mice. Infect Immun (2000) 68(7):4075–83. doi: 10.1128/IAI.68.7.4075-4083.2000 PMC101698 10858224

[48] Flores-Garcia Y Rosales-Encina JL Satoskar AR Talamas-Rohana P. IL-10-IFN-Gamma Double Producers CD4+ T Cells Are Induced by Immunization With an Amastigote Stage Specific Derived Recombinant Protein of Trypanosoma Cruzi. Int J Biol Sci (2011) 7(8):1093–100. doi: 10.7150/ijbs.7.1093 PMC3174386 21927578

